# Combined Robot-Assisted Radical Cystectomy and Robot-Assisted Nephroureterectomy for Synchronous High-Risk Upper Urinary Tract and Bladder Cancer: A Single-Center Retrospective Study and Review of the Literature

**DOI:** 10.3390/jcm15093411

**Published:** 2026-04-29

**Authors:** Nikolaos Kostakopoulos, Konstantinos Evmorfopoulos, Gianluca Maresca, Grigorios Athanasiadis, Athanasios Kostakopoulos, Konstantinos Dimitropoulos

**Affiliations:** 1 1st Department of Urology, Metropolitan General Hospital, 15562 Athens, Greece; 2 Department of Urology, General University Hospital of Larissa, 41110 Larissa, Greece; 3 Department of Urology, Ninewells Hospital, NHS Tayside, Dundee DD2 1UB, UK; 4 Department of Urology, Aberdeen Royal Infirmary, NHS Grampian, Aberdeen AB25 2ZN, UK

**Keywords:** robot, robotic cystectomy, robotic nephroureterectomy, combined cystectomy and nephroureterectomy

## Abstract

**Background/Objectives**: Simultaneous robotic-assisted radical cystectomy (RARC) and nephroureterectomy (RARNU) offers a minimally invasive alternative to open approach for patients with synchronous bladder and upper urinary tract cancers, as well as in selected benign conditions. This study presents our single-center experience and also includes a review of the relevant literature. **Methods**: All patients undergoing combined RARC and RARNU between 2016 and 2023 were retrospectively identified. Clinical and demographic data—including preoperative pathology, operative and re-docking times, estimated blood loss, complications (Clavien–Dindo system), surgical margins, recurrence, morbidity, and follow-up—were collected. A rapid review of the literature was also conducted. **Results**: From 2016 to 2023, 10 patients (mean age 67.4 years, range 56–77) underwent combined RARC and RARNU for upper/lower tract urothelial malignancy. Mean re-docking time was 68.2 min (range 51–100), mean operative time 524.5 min (range 380–690), and mean blood loss 427 cc (range 75–1170). A Pfannenstiel incision was used for en bloc specimen extraction, with no complications or incisional hernias. One case was converted to open surgery, and two required extracorporeal diversion. Postoperatively, five Grade 2 complications were reported, along with one Grade 3, and one Grade 5. All surgical margins were negative. Mean hospital stay was 11.5 days (range 5–29). At a mean follow-up of 21.7 months, one patient had become dialysis-dependent and one had experienced recurrence requiring further surgery. The review of the literature included 74 patients with comparable outcomes. **Conclusions**: Combined RARC and RARNU is a feasible, minimally invasive option for selected patients. Although technically demanding, it offers acceptable safety and should be performed in high-volume, specialized centers.

## 1. Introduction and Objectives

Urothelial bladder carcinoma is the sixth most common malignancy in the United States; specifically, it accounts for 10% of male and 4% of female neoplasms. The occurrence of synchronous high-risk, recurrent or muscle-invasive bladder cancer and upper urinary tract malignancy is rare, affecting fewer than 3% of patients. In some centers the incidence of simultaneous upper-tract and bladder urothelial carcinomas has been reported to be around 2%, with almost half of the bladder tumors being muscle-invasive [1,2].

Surgical management with simultaneous radical cystectomy and nephroureterectomy is the recommended approach in these cases. Furthermore, some benign conditions such as synchronous dialysis-dependent end-stage renal disease or non-functioning kidney and contracted bladder or severe bladder dysfunction can also be indications for removal of the bladder and kidney at the same procedure [1,2]. Moreover, multifocal presentation of urothelial carcinomas is very common among hemodialysis patients; consequently, there is a greater likelihood of a need for combined procedures [1].

Combined radical cystectomy and nephroureterectomy provides significant advantages for patients. Primarily, these combined procedures eliminate the need for multiple operations that require general anesthesia, resulting in reduced morbidity. Additionally, the combined surgical approach can potentially lower overall costs and shorten the recovery period [3].

A few surgical teams have published their experiences in performing open or laparoscopic en bloc cystectomy and nephroureterectomy [4,5]. However, the introduction of advanced robotic-assisted surgical systems in recent years has improved ergonomics and reduced the morbidity associated with the procedure. In addition, robotic-assisted radical cystectomy (RARC with totally intracorporeal urinary diversion) is becoming increasingly popular among urological surgeons. Therefore, combined robotic-assisted radical cystectomy (RARC) and robotic-assisted nephroureterectomy (RARNU) procedures are increasingly being adopted globally, mainly because of the potential lower rates of perioperative complications and shorter hospital stays [5,6,7,8]

This study presents our departmental experience with simultaneous RARNU and RARC for urothelial cell cancers and discusses the relevant literature. We also present the potential benefits of the robotic approach, compared to open combined surgery, which are reported in the available studies, in association with perioperative care and safety.

## 2. Materials and Methods

A retrospective chart review was conducted after approval was obtained from the Institutional Review Board. All consecutive cases of combined robot-assisted radical cystectomy (RARC) and robot-assisted nephroureterectomy (RARNU) performed in our department between 2016 and 2023 were identified. Relevant demographic and clinical data were collected retrospectively, including preoperative histopathology, operative and re-docking times, estimated blood loss, intraoperative and postoperative complications (classified according to the European Association of Urology [EAU] Intraoperative Adverse Incident Classification System and the Clavien–Dindo grading system, respectively), margin status, morbidity, and duration of follow-up.

In parallel, a narrative literature review was undertaken to contextualize our institutional outcomes. A comprehensive search of PubMed and the Cochrane Library databases was performed, using the following associated keywords: [(Robotic nephroureterectomy) AND (Robotic cystectomy) AND (robot)] OR [(combined cystectomy and nephroureterectomy)], in adherence to the PRISMA (Preferred Reporting Items for Systematic Reviews and Meta-Analyses) guidelines. The search aimed to identify studies reporting combined minimally invasive RARC and RARNU procedures. Duplicates, case reports, studies that did not involve simultaneous or combined robotic/laparoscopic approaches, and studies not written in English were excluded. Studies meeting the inclusion criteria were assessed for eligibility and included in the final analysis.

**Figure 1:**
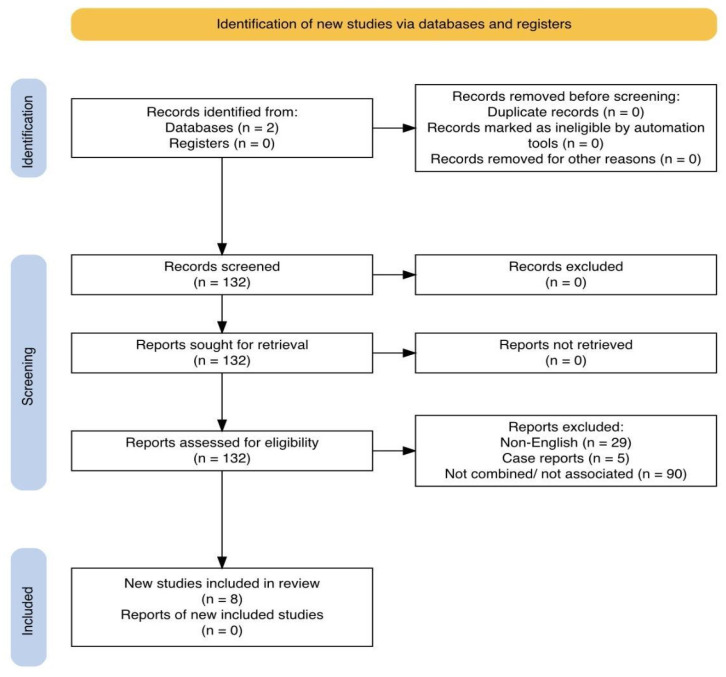
PRISMA flow diagram.

The PRISMA flow diagram detailing the study selection process is presented in Figure 1.

## 3. Results

### 3.1. Cohort Study Results

Between 2016 and 2023, 10 patients (mean age 67.6 years, range 56–77) were offered RARC and RARNU for upper/lower tract urothelial malignancy in our department. All patients were preoperatively treated with neoadjuvant chemotherapy for previously diagnosed muscle-invasive urothelial bladder tumor. A dedicated upper urinary tract robotic surgeon performed the RARNU, and a dedicated pelvic robotic surgeon performed the RARC, with extended lymph node dissection and urinary diversion which for all patients was an ileal conduit formation. With a mean re-docking time of 68.2 min (range 51–100 min), mean operative time was 524.5 min (range 380–690 min) and mean blood loss was 427 cc (75–1170 cc).

Indocyanine green (ICG) 25 mg in 10 mL distilled water with 2 mL solution intravenously was administered prior to ureteroileal anastomosis, to identify the vascularity of the ureteric distal end and thus prevent postoperative ureteroileal strictures.

Moreover, the bowel was handled minimally, using Cadiere forceps and vicryl 2-0 stay sutures which were cut short and clipped with Hem-o-Lok clips so that the bowel was not touched directly.

**Figure 2:**
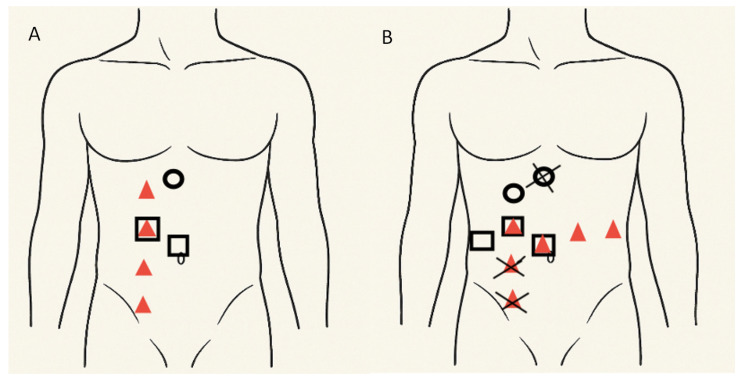
Port placement for RANU (**A**) and RARC (**B**). The squares indicate 12 mm ports. The circles indicate 5 mm assistant ports. The triangles indicate the robotic ports (8 mm). Cross marks indicate port holes that were not used in RARC.

A Pfannenstiel incision was used to extract the entire specimen en bloc at the end of the procedure, including the urinary bladder, ureter, and kidney, with a separate specimen bag used for lymph nodes. No complications or incisional hernias were observed. The two middle initial ports of the RARNU were repurposed as assistant and robotic ports during the RARC phase, while the most caudal RARNU incision was extended to facilitate the Pfannenstiel specimen extraction. Three additional robotic trocars were inserted for the RARC component. Figure 2 shows the trocar arrangement for RARNU and RARC.

One case was partly converted to open (part of RNU), and two cases required extracorporeal ileal conduit diversion. Five Grade 2 complications (50%), one Grade 3 complication (10%) and one Grade 5 complication (10%) were recorded postoperatively. All margins were clear, and mean hospital stay was 11.5 days (range 5–29 days). At mean follow-up of 21.67 months (range 3–50 months) one patient (10%) had become dialysis-dependent because of a recurrence that required further surgical intervention (radical urethrectomy and contralateral nephroureterectomy). During the same follow-up period, no patient received adjuvant systematic treatment apart from the patient with the recurrence who was treated with a combination of immunotherapy and chemotherapy by the oncologists.

### 3.2. Rapid Review of the Literature

Our rapid review of the literature identified 74 patients who underwent combined minimally invasive radical cystectomy and nephroureterectomy in the eight case series that were identified and included in our study. In five studies, both parts of the procedure were done robotically (53 patients). In one study, both the cystectomy and nephroureterectomy were done laparoscopically. Two case series used the laparoscopic approach for the upper tract and the robotic approach for the bladder.

There was no need for conversion to open or intraoperative complications reported in any of the studies. In most of the case series the reason for combining RARC and RARNU was simultaneous bladder and upper urinary tract urothelial carcinoma. However, in three of the included studies the initial diagnosis in some patients was nonfunctioning kidney or end-stage renal disease (15% median of all eight studies), although there were no cases of preoperative benign bladder diagnosis. Furthermore, there were two cases of urothelial bladder cancer with squamous differentiation, and one squamous cell carcinoma of the bladder.

One study compared open and robotic simultaneous approaches. This study showed one case of positive surgical margins in the robotic arm, while there were three recurrences among the open cases and two among the robotic cases. One patient from the open series died within 6 months of surgery. Moreover, three patients from the open arm of the study had more than three Clavien–Dindo complications, in comparison to none from the robotic arm. In total, only six patients (8.1%, 6/74) that underwent simultaneous RARC and RARNU (or laparoscopic either part) had high-grade (>3) complications, including two deaths, at the postoperative follow-up (<90 days). Only nine of the patients who underwent robotic procedures were transfused (12.16%, 9/74), while almost all patients subject to the open simultaneous approach needed transfusion (94%, 16/17). The exact number of units was not reported in all studies, but one patient was reportedly transfused with 2 units of blood.

During postoperative follow-up, there was one case of metachronous urothelial cell cancer of the contralateral kidney 24 months after the initial procedure; there were also two patients with tumor progression, at 4 and 62 months, as well as three cases of positive surgical margins The results of the studies are shown in Table 1.

## 4. Discussion

The present study evaluates the feasibility, safety, and outcomes of combined robot-assisted radical cystectomy (RARC) and robot-assisted radical nephroureterectomy (RARNU) in patients with synchronous high-risk upper urinary tract and bladder urothelial carcinoma. Our findings, contextualized within the existing body of literature, contribute to a growing body of evidence supporting this complex, minimally invasive approach.

The presence of concurrent muscle-invasive urothelial cell carcinoma (UCC) or recurrent high-risk superficial UCC of the bladder and synchronous UCC of the upper urinary tract is uncommon [1,2,10,11,12]. A simultaneous radical nephroureterectomy and radical cystectomy as a one-step procedure can be performed to reduce anesthetic risk and morbidity among patients. There are a very small number of available studies reporting on combined unilateral or bilateral nephroureterectomy and radical cystectomy; these mainly consist of small case series and case reports.

Our series demonstrates that simultaneous RARC and RARNU is technically feasible in specialized centers with an experienced multidisciplinary robotic team. The mean operative time (524.5 min) and mean estimated blood loss (427 cc) observed in our cohort are consistent with previously published case series, where operative times ranged from 252 to 560.8 min [1,8] and blood loss from 220 to 775 cc [2,6]. The need for re-docking and coordinated teamwork between upper-tract and pelvic surgeons is reflected in the operative metrics, but did not translate into increased intraoperative complications or conversion rates, with only one conversion to open surgery in our series. The use of a Pfannenstiel incision for en bloc specimen extraction proved effective, with no incisional hernias or wound complications, mirroring the safety profile reported in other studies. Furthermore, the use of indocyanine green (ICG) to recognize the vascularity of the remaining ureter before ileal conduit diversion in our series eradicated postoperative ureteric stricture incidences.

All patients in our series had negative surgical margins, with only one recurrence requiring further surgical intervention during follow-up. These results are in line with published data, where positive margin rates remain low, and recurrence rates are not increased compared to open or staged procedures. A recent meta-analysis reported 2-year and 5-year overall survival rates of 68% and 44%, respectively, for simultaneous radical cystectomy and nephroureterectomy, with a 2-year progression-free survival rate of 91% [13]. These outcomes underscore the aggressive nature of synchronous urothelial carcinoma, but do not suggest that cancer control is inferior with the combined robotic approach.

A recent systematic review and comparative analysis aimed to investigate the indications, clinical outcomes, and procedure safety of simultaneous RC and RNU [10]. From a total of 28 studies including 947 patients, it was found that 32,9% of patients underwent laparoscopic surgery while 25.2% underwent robot-assisted surgery [10]. The outcomes of the different surgical approaches could not be compared in the systematic review, because of study heterogeneity and limitations; however, robotic-assisted surgery was found to be associated not only with a lower blood transfusion rate but also with longer operative times when compared to open surgery, while all remaining outcomes were found to be similar [8,10].

Simultaneous RC and RNU has been shown to involve a higher rate of intraoperative and postoperative complications and an increased risk of re-operation, when compared to RC alone, and thus it is a treatment option that should be cautiously used in well-informed patients who are relatively fit or in whom the expected benefits outweigh the procedure-related risks [10].

Simultaneous RARC and RARNU provides a single-stage solution for patients with multifocal or synchronous high-risk urothelial malignancies, reducing the need for multiple anesthetics and hospitalizations. This is particularly advantageous for patients with significant comorbidities, end-stage renal disease, or non-functioning kidneys, where minimizing perioperative risk is crucial [10]. Moreover, the robotic approach has oncological and functional results comparable with those obtained using the traditional open approach, but with lower postoperative morbidity and mortality [14,15]. Moreover, contemporary robotic surgery such as robotic nephroureterectomy has significantly improved outcomes and achieved lower complication rates; this can usually be attributed to the greater experience of robotic surgeons and the higher volume of robotic cases in tertiary hospitals [15]. However, careful patient selection and treatment in specialized centers remain essential to optimize outcomes and safety.

The retrospective design and small sample size limit the generalizability of our findings, as is the case with most published series. Hence, comparisons have been made with studies of similarly small sample size. In addition, selection bias in our case series was high because all of the patients had relatively low or intermediate levels of comorbidities, so that they could be fit enough for combined robotic surgery (Charlson comorbidity index lower than 4), which is associated with a lower risk of complications in the literature [16].

Larger, multi-center prospective studies with longer follow-ups are necessary to better define the oncological efficacy, complication rates, and optimal patient selection criteria for combined minimally invasive approaches. The combination of specialized surgical team experience in robotic combined procedures as well as the inclusion of more patients with a larger range of comorbidities and oncological factors, will clarify and evaluate the safety profile of combined RARC and RARNU in the future, and standardize the technique in order for it to be officially included in international clinical guidelines [10,13].

## 5. Conclusions

In summary, our experience and the available literature both suggest that combined RARC and RARNU is a relatively safe and promising option for selected patients with synchronous high-risk upper and lower urinary tract urothelial carcinoma. The retrospective results suggest that the robotic approach offers perioperative advantages without compromising oncological outcomes in specialized, high-volume centers, though further prospective studies with longer follow-up are warranted to confirm these findings in larger cohorts.

## Figures and Tables

**Table 1:** Results of studies with combined robot-assisted cystectomy (RARC) and robot-assisted nephroureterectomy (RARNU). LRC—laparoscopic radical cystectomy, LRNU—laparoscopic radical nephroureterectomy, RNU—radical nephroureterectomy.

Study	Journal/Year	Number of Patients/Age	Approach	Intraoperative Complications/Conversion to Open	Operative Time	EBL (Estimated Blood Loss)	Hospital Stay	Postoperative Complications (Clavien–Dindo ≥ 3)	Follow-Up/Recurrences
**Kostakopoulos et al. (our study)**	**2016–2023**	**10/67.6 (mean)**	**RARC & RARNU**	**1**	**524.5 min (range 380–690 min)**	**427 cc (75–1170 cc)**	**11.5 days (range 5–29 days)**	**(1) 3b (1) 5**	**21.67 months (range 3–50 months)/1**
**Ou et al. [1]**	J Endour 2011	8/66.9 (mean)	RARC & RARNU	0	group II (252.5 ± 35.0 min) vs. group I (360 ± 25.8 min) (mean)	group II (332.5 ± 53.8 mL) vs. group I (660 ± 137.4 mL) (mean)	7.75 days (mean)	no	28.1 months (mean)/0
**Barros et al. [2]**	Int Braz J Urol 2008	8/76.5 (median)	LRC & LRNU	0	540 min (median)	755 mL (median)	7.5 days (median)	(1) 4a (fistula) (1) 5 (death)	9 months/1 metastases/1 death
**Pisipati et al. [3]**	Can Urol Assoc J. 2014	6/71 (median)	RARC & RARNU	0	300 min (mean)	416.7 mL (mean)	10.2 days (mean)	no	not reported
**Yajima et al. [5]**	Curr Urol. 2021	3/75 (median)	RARC & LRNU	0	435 min (Median)	377 mL (median)	26 days (median)	(1) 3b (incisional hernia)	8 months (median)/0
**Buse et al. [6]**	BMC Urol 2021	19/73 (mean)	RARC & RARNU	0	324 min (Median)	220 mL (median)	not reported	(1) 3b (post op hernia) (1) 5 (death/pulmonary embolism)	23 months (median)/1 contralateral kidney recurrence & 3 deaths
**Buse et al. [7]**	J Robot Surg 2016	11/74 (mean)	RARC & RARNU	0	287 min (median)	235 mL (median)	15 days (median)	(1) 3b (port hernia)	7 months (median)/1 contralateral kidney recurrence
**Kamei et al. [8]**	Asian J Endosc Surg 2022	10 & 17/69.2 ± 6.2 (RARC), 70.0 ± 8.4 (open)	10 RARC & lapRNU vs. 17 OPEN RC & RNU	0	560.8 ± 80.7 min (rarc), 487.1 ± 98.7 (open)	291.0 ± 268.8 mL (rarc), 2790.0 ± 1341.4 mL (open)	17.8 ± 4.2 (RARC), 30.7 ± 14.7 (open)	(3/17) Grade ≥ 3 (open) (1/17) 5 (death)	6 months/Recurrence: 2 robotic, 3 open. Mortality: 1 open
**Lin et al. [9]**	J Chin Med Assoc 2014	9	RARC & RARNU	0	295 min (mean)	470 mL (mean)	not reported	no	not reported

## Data Availability

The original contributions presented in this study are included in the article. Further inquiries can be directed to the corresponding author.
